# Knockout of *ovary serine protease* Leads to Ovary Deformation and Female Sterility in the Asian Corn Borer, *Ostrinia furnacalis*

**DOI:** 10.3390/ijms242216311

**Published:** 2023-11-14

**Authors:** Porui Zhang, Zuerdong Jialaliding, Junwen Gu, Austin Merchant, Qi Zhang, Xuguo Zhou

**Affiliations:** 1 College of Plant Protection, Shenyang Agricultural University, Shenyang 110866, China; zprzpr1997@gmail.com (P.Z.); zrd0527@stu.syau.edu.cn (Z.J.); junwengu2022@stu.syau.edu.cn (J.G.); 2 Department of Entomology, University of Kentucky, Lexington, KY 40546, USA; ajme232@g.uky.edu

**Keywords:** *Ostrinia furnacalis*, *Ovarian serine protease*, CRISPR/Cas9 gene editing, SIT

## Abstract

Oogenesis in insects is a carefully orchestrated process, facilitating the formation of female gametes, which is regulated by multiple extrinsic and intrinsic factors, including *ovary serine protease (Osp).* As a member of the serine protease family, *Osp* is a homolog of *Nudel*, a maternally required protease defining embryonic dorsoventral polarity in *Drosophila*. In this study, we used CRISPR/Cas9-mediated mutagenesis to functionally characterize *Osp* in the Asian corn borer, *Ostrinia furnacalis,* a devastating maize pest throughout Asia and Australia. Building on previous knowledge, we hypothesized that knockout of *Osp* would disrupt embryonic development in *O. furnacalis* females. To examine this overarching hypothesis, we (1) cloned and characterized *Osp* from *O. furnacalis*, (2) designed target sites on exons 1 and 4 to construct a CRISPR/Cas9 mutagenesis system, and (3) documented phenotypic impacts among *O. furnacalis Osp* mutants. As a result, we (1) examined the temporal-spatial expression profiles of *OfOsp*, which has an open reading frame of 5648 bp in length and encodes a protein of 1873 amino acids; (2) established *O. furnacalis Osp* mutants; and (3) documented recessive, female-specific sterility among *OfOsp^F^* mutants, including absent or deformed oviducts and reduced fertility in female but not male mutants. Overall, the combined results support our initial hypothesis that *Osp* is required for embryonic development, specifically ovarian maturation, in *O. furnacalis* females. Given its substantial impacts on female sterility, *Osp* provides a potential target for the Sterile Insect Technique (SIT) to manage *Lepidoptera pests* in general and the species complex *Ostrinia* in particular.

## 1. Introduction

The Asian corn borer, *Ostrinia furnacalis* (Guenée) (*Lepidoptera*: *Pyralidae*), is an important corn pest that significantly threatens crop yields and quality [1]. The geographic range of *O. furnacalis* extends throughout Asia, the Western Pacific, and Australia [2,3]. The larvae of *O. furnacalis* mostly infest the ears and leaves of corn and bore tunnels in corn stems, resulting in crop losses of up to 30% in cases of severe infestation. Damage caused by *O. furnacalis* can lead to fungal contamination, which further influences the quality of fresh corn [4]. In addition to corn, *O. furnacalis* infects a variety of other hosts including soybeans, wheat, cotton, sorghum, and millet [5,6]. Control of *O. furnacalis* relies on chemical pesticides and some biological control agents, such as parasitic wasps [7] and the insecticidal Bt toxin [8,9]. However, overuse of pesticides is associated with the development of resistance and environmental concerns [10,11]. Thus, an alternative strategy to control *O. furnacalis*, the most destructive pest of corn, is urgently needed.

Successful application of the Sterile Insect Technique (SIT) is dependent on the release of sterile insects into the field, which mate with wildtype insects and either produce abnormal offspring or eliminate offspring altogether, thereby controlling the target insect [12,13]. The SIT includes radiation-based (rSIT), microbe-mediated (mSIT), and genetic-based (gSIT) methods [14,15,16]. Specifically, rSIT involves the release of radio-sterilized males into the wild as an environmentally friendly means of suppressing or eradicating insect pests [5,17,18]. mSIT has been most successful as a means of inducing cytoplasmic incompatibility via wolbachia and gut microbiota [19,20]. gSIT utilizes genome editing tools such as the CRISPR/Cas9 system to induce adult sterility [16,21,22,23,24].

Serine proteases work as proteolytic enzymes that modulate a variety of physiological processes, such as digestion, signal transduction, and defense reactions [25,26], and are also involved in sperm differentiation and function in mammals [27,28]. In insects, serine proteases are required for post-mating responses in female *D. melanogaster* [29]. Recent studies in lepidopteran insects have also verified the vital role of serine proteases in male sterility and female reproductivity [30,31]. Ovarian serine protease (*Osp*) is homologous to *Nudel* in *D. melanogaster* [32]. *Osp* is expressed by ovarian follicular cells during mid and late vitellogenesis in *Bombyx mori* [31]. Nudel protein is expressed as an extracellular matrix-like molecule that may be anchored in the vitelline membrane [33]. *Nudel* is responsible for specifying embryonic dorsoventral polarity, development of embryogenesis, and oogenesis in *D. melanogaster,* as well as increasing the structural integrity of the egg [33]. Mutations in *Nudel* have weakened the structural integrity of eggs, hindered development, and ultimately led to embryonic death, resulting in female infertility [34]. In *B. mori*, Osp protein shares 65% of its identity with the homologous *Drosophila* Nudel protein, and mutagenesis of *Osp* leads to abnormal oogenesis and female sterility that can be inherited by offspring, indicating the conserved function of Osp protein in lepidopteran insects [31].

In this study, we aimed to functionally characterize the role of *O. furnacalis Osp*, *OfOsp*, in ovarian development and reproduction. We hypothesized that *OfOsp* disrupts embryonic development in *O. furnacalis* females. To examine this hypothesis, we (1) cloned and characterized *OfOsp*, (2) knocked out *OfOsp* in *O. furnacalis* using the CRISPR/Cas9-mediated gene-editing system, and (3) documented the morphological and physiological consequences caused by *OfOsp* mutagenesis.

## 2. Results

### 2.1. Cloning and Characterization of OfOsp

#### 2.1.1. Cloning of *OfOsp*

The coding sequence of *OfOsp* (XM_028323031) was obtained by checking BLAST *B. mori Osp* (GenBank: NM_001043703.1) against the *O. furnacalis* genome [35]. As a result, a homologous Osp protein (XP_028178832.1) that shares 70.61% of its identity with *B. mori* Osp (NP_001037168.1) was identified. *OfOsp* is 5648 bp in length and encodes 1873 amino acids. Primers were designed to amplify approximately 1500–2500 bp fragments, and the full-length Osp open reading frame (ORF) was assembled from the resulting sequencing data (Table 1). After sequencing, the full length *OfOsp* ORF was obtained and was used to design sgRNAs.

#### 2.1.2. Phylogenetic Analysis

We analyzed the homologous sequences of Osp proteins selected from 10 different insect species to determine their evolutionary conservation against *O. furnacalis*, including the lepidopterans silkworm, *B. mori*; the cotton bollworm, *Helicoverpa armigera*; the diamondback moth, *Plutella xylostella*; and the fall armyworm, *Spodoptera frugiperda*, as well as other non-lepidopterans fruit flies, *D. melanogaster*; parasitic wood wasps, *Orussus abietinus*; wheat stem sawflies, *Cephus cinctus*; burying beetles, *Nicrophorus vespilloides*; red flour beetles, *Tribolium castaneum;* and green peach aphids, *Myzus persicae*. Not surprisingly, *OfOsp* shared the closest relationship with fellow lepidopterans, including *B. mori*, *P. xyllostella*, *S. frugiperta*, and *H. armigera*. *Osp* in *B. mori* (NP_001037168.1) encodes 1801 amino acids with a 6273 bp ORF, while the length of *Osp* in *H. armigera* (XP_021187246.1) is 6447 bp, and it encodes 2149 amino acids; the length of *Osp* in *P. xylostella* (XP_011552048.1) is 5982 bp, and it encodes 1994 amino acids; and the length of *Osp* in *S. frugiperda* is 6362 bp, and it encodes 2034 amino acids (Figure S1).

#### 2.1.3. Temporal–Spatial Distribution of *OfOsp*

**Figure 1:**
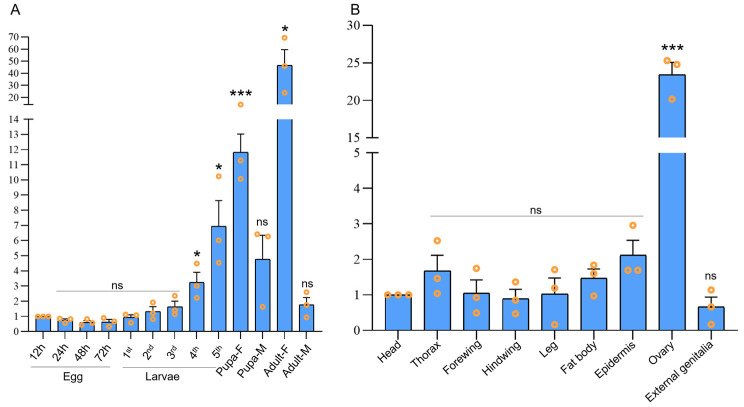
The temporal and spatial distributions of *OfOsp*. Data are shown as the mean ± SEM (*n* = 3). (**A**) The temporal distribution of *OfOsp* in eggs at 12 h, 24 h, 48 h and 72 h, larvae at instars 1 to 5, female and male pupae, and female and male adults was examined. (**B**) The spatial expression analysis from tissues of heads, thoraxes (with no legs and wings), legs, forewings, hindwings, abdominal epidermis, fat bodies, ovaries, and external genitalia was examined. One-way ANOVA in SPSS statistics 22 was used for data analysis. “ns” stands for “not significant” (*p* > 0.05). The asterisks (* and ***) indicate significant differences (*p* < 0.05 and *p* < 0.001).

In *S. litura, Osp* exhibits relatively high expression in female pupae and adults, with tissue-specific expression in the ovaries [31]. In *O. furnacalis*, the transcript levels of *OfOsp* in fourth-instar larvae, fifth-instar larvae, female pupae, and female adults were significantly higher compared to those in the early 12 h old eggs post-laying (*p* < 0.05). The highest transcript level of *OfOsp* was observed in adult females, which was about 46-fold higher than that in the egg stage, and this was followed by female pupae (Figure 1A), suggesting that *OfOsp* plays an important role in the formation of ovaries in pupae and adults. As for the spatial distribution, the transcript level of *OfOsp* was approximately 23-fold higher in the ovaries of adult females than in adult heads (Figure 1B). These temporal and spatial expression patterns support the potential role of *OfOsp* in ovarian development during the pupal and adult stages (Figure 1).

### 2.2. Mutagenesis of OfOsp Using CRISPR/Cas9

**Figure 2:**
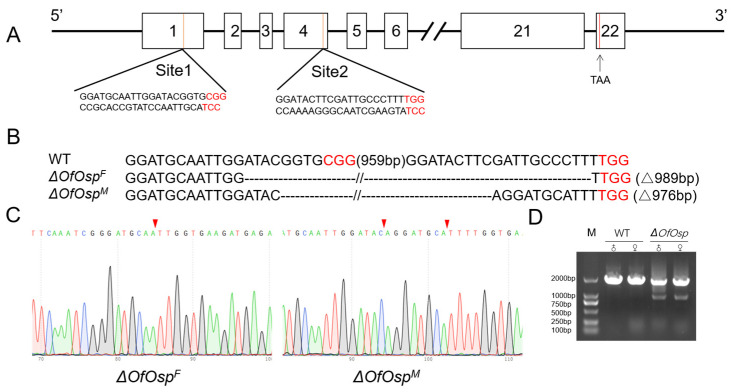
Design of sgRNAs and mutagenesis of *OfOsp* using CRISPR/Cas9 system. (**A**) The design of sgRNAs targeting exons 1 and 4 of *OfOsp*. (**B**) The number of base pairs deleted in the edited sequences (indicated in parentheses). PAM sequences are highlighted in red. “Δ989 bp” refers to the number of base pairs deleted after gene knockout. (**C**) Target sites of *OfOsp* using CRISPR/Cas9 system in male and female *O. furnacalis* (labeled with red arrows). (**D**) Fragments corresponding to Osp mutants and WT in genomic DNA amplified using PCR and resolved with agarose gel electrophoresis.

*OfOsp* contains 22 exons and 21 introns (Figure 2A). CRISPR/Cas9-mediated mutagenesis was used to investigate the functionality of *OfOsp* in *O. furnacalis*. Two sgRNAs targeting two different positions on exons 1 and 4 of *OfOsp* were designed, and the positions of the sgRNAs on these exons are illustrated in Figure 2A. We injected 347 eggs with Cas9 protein and the two sgRNAs and obtained a 61.64% hatching rate and a 28.42% mutation rate in the treated eggs. All insertions or deletions resulted in premature stop codons, leading to truncation or loss of function of the OfOsp protein. The female mutants had a 989 bp fragment missing from exons 1 to 4, whereas male mutants showed a deletion of 976 bp from exons 1 to 4, producing an N-terminal truncated OfOsp protein (Figure 2B). DNA from molted *OfOsp* mutants and wildtype individuals was extracted and sequenced, demonstrating that *OfOsp* was successfully mutated (Figure 2C). Using primers flanking the two target sites at exons 1 and 4 in mutant and WT adults, the resultant banding pattern further indicated that *OfOsp* was successfully mutated (Figure 2D).

### 2.3. Phenotypic and Physiological Impacts Experienced by OfOsp Mutants

#### 2.3.1. Phenotypic Impacts on Internal Genitalia

**Figure 3:**
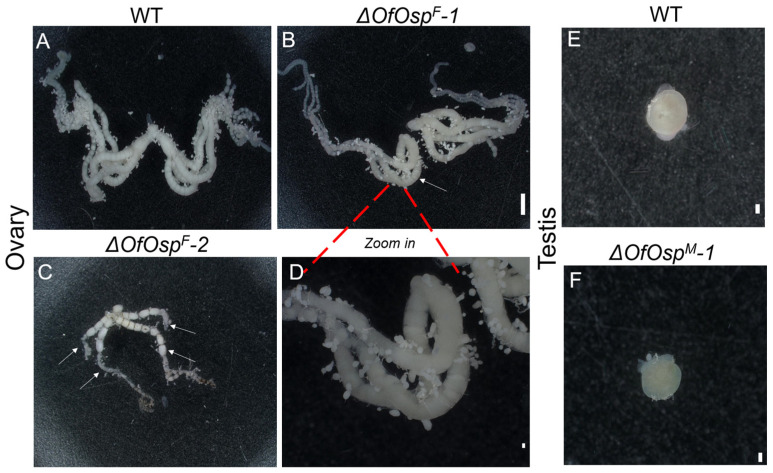
Malformations of ovaries and testes in adult *OfOsp* mutants. The ovaries of wildtype *O. furnacalis* and *OfOsp^F^* and *OfOsp^M^* mutant individuals were dissected on the third day post-eclusion (PAE3). (**A**) Ovary dissected from female wildtype. (**B**) Ovary dissected from female *OfOsp*-1 mutant. (**C**) Ovary dissected from female *OfOsp*-2 mutant. (**D**) Enlargement of the oviducts from *OfOsp*-1 mutant. (**E**) Testes dissected from male wildtype. (**F**) Testes dissected from *OfOsp*-1 male mutant. White arrows indicate defects in the ovaries. Scale bar: 1 mm.

The genitalia of wildtype and *OfOsp* mutant *O. furnacalis* adults were dissected to observe the phenotypic changes associated with mutagenesis. The ovaries and testes of mutant *O. furnacalis* adults, together with those from wildtype groups, were dissected on the third day post-eclosion (Figure 3). Wildtype females possessed ovaries supplemented with four pairs of oviducts that were localized on two sides (Figure 3A). As expected, female *OfOsp^F^* mutants had severe malformations of the ovaries, having one oviduct missing on one side, leaving seven oviducts remaining (Figure 3B), with a closeup view of the oviducts shown in Figure 3D. Additionally, we observed only two pairs of oviducts that were shortened relative to the wildtype females and generated fewer or smaller eggs within the oviducts of *OfOsp^F^* compared to the wildtypes (Figure 3C). The ovaries of *OfOsp^F^* mutants also showed fused and shortened oviducts that either produced fewer eggs or failed to develop normally (Figure 3C). Male *OfOsp* mutants presented normal phenotypes with no phenotypic changes observed compared to the wildtypes (Figure 3E,F).

#### 2.3.2. Effects of *OfOsp* Mutagenesis on Fertility and Fecundity

**Figure 4:**
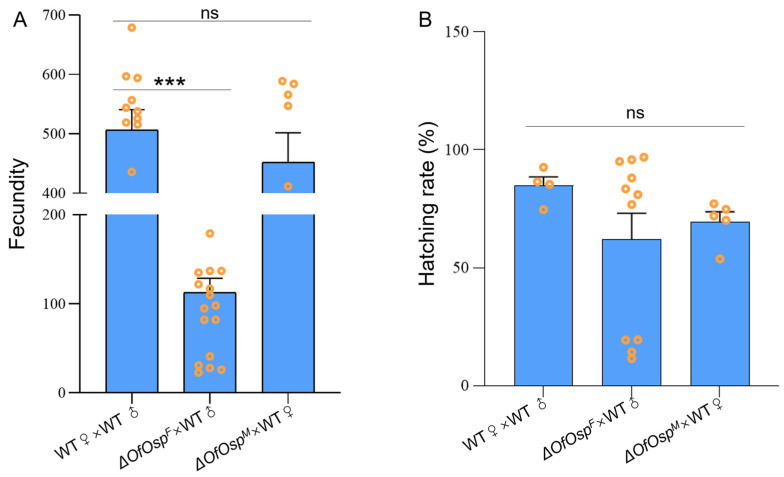
Physiological impacts of *OfOsp* mutagenesis. (**A**) Fecundity of adult mutants of *OfOsp* paired with opposite gender of wildtype are shown. (**B**) The hatching rates of eggs laid by *OfOsp* female and *OfOsp* male comparing with wildtypes. Data are shown as the mean ± SEM (*n* = 5–20), and one-way ANOVA in SPSS statistics 22 was used for data analysis. “ns” stands for “not significant” (*p* > 0.05). The asterisks (***) indicate significant differences (*p* < 0.0001). The orange circles indicate the value of each replicate recorded for fecundity and hatching rate.

Ovary development is important for the fertility and fecundity of female adults. To investigate if *OfOsp* mutagenesis leads to adult sterility, the fecundity and hatching rates of both mutant and wildtype *O. furnacalis* were recorded continuously for ten days after pairing individuals with wildtype individuals of the opposite sex. In wildtype pairings, females laid an average of 483 ± 34 eggs within the ten-day period. *OfOsp^F^* mutants laid an average of 113 ± 16 eggs within the ten-day period, which is significantly decreased compared to wildtype females. Wildtype females paired with *OfOsp* mutant males laid 340 ± 65 eggs within the ten-day period, which was not significantly different from pure wildtype pairings (Figure 4A). The hatching rate of eggs laid by *OfOsp* female and male mutants showed no significant differences in comparison to the wildtypes (Figure 4B).

### 2.4. Pleiotropic Effects of OfOsp Mutagenesis

**Figure 5:**
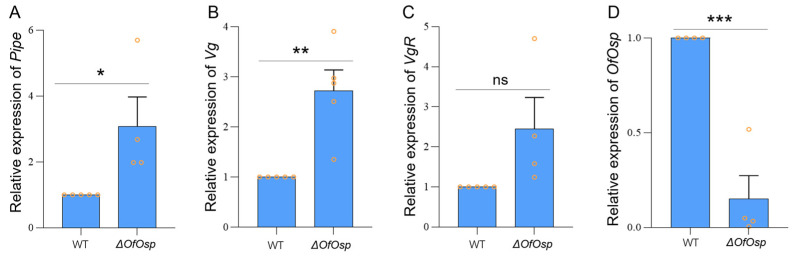
Relative expressions of *Pipe*, *Vg*, *VgR,* and *OfOsp* after *OfOsp* mutagenesis. The relative transcript levels of *Pipe* (**A**), *Vg* (**B**) and *VgR* (**C**)*,* and *OfOsp* (**D**) were examined in ovaries of *OfOsp* female mutants comparing with that in the female ovaries of wildtype . Data are shown as the mean ± SEM (*n* = 4–5), and one-way ANOVA in SPSS statistics 22 was used for data analysis. The orange circles indicate values of relative expression of *Pipe* (**A**), *Vg* (**B**) and *VgR* (**C**)*,* and *OfOsp* (**D**) normalized to *OfRPS3,* the internal reference gene calculated by 2^-ΔΔCq^ method. “ns” stands for “not significant” (*p* > 0.05). The asterisks (*, ** and ***) indicate significant differences (*p* < 0.05, *p* < 0.01 and *p* < 0.001) between mutants and wildtypes.

Given that *Vitellogenin (Vg)* and *Vitellogenin receptor (VgR)* are associated with the process of vitellogenesis in insects [36], we examined the relative transcript levels of *Vg* and *VgR* in the ovaries of *O. furnacalis* after *OfOsp* mutagenesis. The relative transcript level of *pipe* was also examined in *OfOsp* mutants, where *pipe* was significantly upregulated in *OfOsp^F^* mutants by 3.08-fold compared to the wildtypes (Figure 5A). The results showed that *Vg* was upregulated by 2.72-fold when normalized to the wildtypes after *OfOsp* mutagenesis (Figure 5B). The relative transcript level of *VgR* was not changed (Figure 5C). In *Drosophila*, *Pipe* together with *Nudel* are required for embryonic dorsal–ventral polarity [37]. We also further verified the transcript levels of *OfOsp* in the ovaries of *OfOsp^F^* mutants, which were significantly decreased compared to the wildtypes (Figure 5D).

## 3. Discussion

### 3.1. Identification and Characterization of OfOsp

*Nudel* is expressed during oogenesis and is essential for egg development after fertilization [33]. In lepidopterans, *Osp* was first analyzed as a homolog to *Drosophila Nudel* in *B. mori* and *S. litura* [31]. Mutagenesis of *OSP* in these two species causes abnormal development in the ovaries and female sterility [31] (Figure S1). Using the published *O. furnacalis* genome [35], we identified a homologous Osp protein that shares 70.61% of its identity with the *B. mori* Osp, and phylogenetic analysis supports that Osp is highly conserved among lepidopteran insects (Figure S1).

*Osp* is highly expressed in the ovaries of female pupae and adults in lepidopteran insects [31]. In our study, temporal, and spatial expression patterns of *OfOsp* were analyzed to show its increased expression in the ovarian tissues of female adults, supporting its essential role in germ line formation and oogenesis during embryonic development (Figure 1A). Temporal expression verified the expression level of *OfOsp* in different developmental stages, showing that *OfOsp* is highly expressed in the fourth- and fifth-instar larvae, female pupae, and female adults of *O. furnacalis* (Figure 1B).

### 3.2. Mutagenesis of OfOsp Using CRISPR/Cas9 Gene Editing System

The specificity and flexibility of the CRISPR/Cas9 system allows precise editing of genes that would benefit strategies for pest management. In the mosquito *Anopheles gambiae*, using CRISPR/Cas9 to function as a gene drive system, three putative female fertility-related genes were identified and targeted to successfully suppress mosquito populations that transmit malaria [32]. *S. frugiperda* was disrupted through embryonic microinjection of Cas9 protein and in vitro-transcribed sgRNAs targeting *doublesex*, leading to impaired ovaries and testes in adults and an inheritable reduction in fertility and fecundity [38]. Our results show that mutagenesis of *OfOsp* results in missing and malformed oviducts, with oviducts in some cases fusing together, disabling their ability to produce normal eggs (Figure 3). In *B. mori*, knockout of *Osp* leads to irregular oviducts and shorter and shrunken oviducts in mutants [31]. Our results have further verified the functional role of *Osp* in ovarian development and oogenesis in lepidopterans.

### 3.3. Phenotypic Impacts among OfOsp Mutants

Regulating and disrupting the secretion of proteins from insect reproductive glands as a means of pest control has become a new strategy in the field of insect infertility technology. Gligorov and colleagues discovered that the absence of *Abd-B* in *D. melanogaster* resulted in disruption of the ability of females to re-mate with males and to lay eggs when mating with mutated male individuals [39]. Similarly, knockout of *Abd-A* and *Ubx* leads to defects in wing formation and reduced reproductivity in *O. furnacalis* [40]. Other potential targets for SIT to control *O. furnacalis* are *Masc* and *doublesex*, the mutagenesis of which leads to reduced fecundity and fertility [24]. Our results have identified an additional target to control *O. furnacalis* via the release of mutant males that carry a mutated *OfOsp* to mate with females in the wild. In this study, we observed the development of abnormal ovaries in female *OfOsp* mutants and normal testes in males (Figure 3). As a result, the fertility of female, not male, mutants is significantly decreased after *OfOsp* knockdown (Figure 4), indicating the functional role of *OfOsp* in ovarian development and oogenesis. This result is inconsistent with the results for *B. mori* and *S. litura*, in which knockdown of *Osp* significantly reduces fertility [31].

Insect oogenesis requires expression of a variety of genes. *Pipe* is required in the somatic tissue of *Drosophila* egg chambers and defines embryonic dorsal–ventral polarity [37]. We first examined the relative expression of *OfOsp* in the dissected ovaries in *OfOsp* mutants and found that knockout of *OfOsp* significantly decreases the expression of *OfOsp* (Figure 5D). Since *Osp* and *Pipe* are products of dorsal-group genes that play key roles in spatially regulating the protease cascade involved in controlling embryonic patterning and polarity in Drosophila [41], we examined the relative transcript levels of *pipe* and found that they were upregulated in *OfOsp^F^* mutants compared to the wildtypes (Figure 5A). Previous studies have reported that the *Pipe* gene is highly expressed in the ovaries of *Drosophila* [37]. Our results have further indicated its complementary role in oogenesis alongside *Osp*.

RNAi targeting *Vg* and *VgR* in *Diaphorina citri* Kuwayama leads to disturbed formation of eggs and suppresses oviposition by adults [42]. We examined the relative transcript levels of *Vg* and *VgR* in the dissected ovaries of *OfOsp* mutants. The transcript level of *Vg* was upregulated 3.04-fold in comparison to the wildtypes (Figure 5), indicating its role in ovary development as complementary to *OfOsp*. Our results are in accordance with the known role of *Vg* as being responsible for the formation of yolk proteins in insect eggs [43]. As *Vg* transcripts accumulated in the ovaries of *Culex quinquefasciatus* [44], the altered upregulation of *Vg* in our study has further verified its essential role in the ovarian development.

## 4. Materials and Methods

### 4.1. Insect Rearing and Sexing

The *O. furnacalis* strain used in this study was collected from corn in Qipanshan County, Shenyang City, Liaoning Province, China (123.471097,41.68383) and was kept in laboratory conditions at a temperature of 25 °C with a relative humidity of 70%, light intensity of 1000 lx, and a photoperiod of 16:8 h L:D. *O. furnacalis* larvae were fed with a standard lepidopteran artificial diet made with yeast extract, wheat bran, vitamin C, sucrose, and agar, as described previously, every two days [38]. Pupae were sexed for pairing post-eclosion, and adults were kept in rectangular-shaped metal rearing cages supplemented with cotton balls, which were soaked with 10% honey water to allow for mating and egg laying.

### 4.2. Cloning and Characterization of OfOsp

#### 4.2.1. Cloning of *OfOsp*

To obtain the coding sequence of *OfOsp* (XM_028323031), we performed a BLAST search using *B. mori Nudel* (GenBank: NM_001043703.1) against the *O. furnacalis* genome in NCBI. Total RNA from a fifth-instar *O. furnacalis* larva was extracted using TRIeasy reagent (Yeasen, China) to amplify the full length of *OfOsp*. The extracted RNA was then reverse transcribed into cDNA using the PrimeScript^TM^ Reverse Transcription Kit (Takara, Japan) using 1µg of total RNA as a template. The full-length sequence of *OfOsp* was 5648 bp in length, encoding 1873 amino acids. Primers were designed to amplify 1500–2000 bp fragments of *Osp* ORF and the full-length sequence was assembled from the resulting sequencing data. After sequencing (Genewiz biotech company, Suzhou, China), the full-length sequence of *OfOsp* was constructed and verified before designing sgRNAs. All primers were designed using primer3 (https://primer3.ut.ee (accessed on 1 May 2000)).

#### 4.2.2. Phylogenetic Analysis of OfOsp

Osp ORFs used in the phylogenetic analysis included the OfOsp identified in this study, and other Osps from *B. mori* (NP_001037168.1), *H. armigera* (XP_021187246.1), *P. xylostella* (XP_011552048.1), *S. frugiperda* (XP_035438721.2), *D. melanogaster* (NM_079223.2), *O. abietinus* (XP_012280703.1), *C. cinctus* (XP_015603565.1), *N. vespilloides* (XP_017777496.1), *T. castaneum* (XP_015840900.1), and *M. persicae* (XP_022174293.1). Osp ORFs were aligned with ClustalW and the resultant phylogenetic tree was constructed using the neighbor-joining (N-J) method using MEGAX. In addition, *p*-distance model was selected for the substitution model. Finally, to assess the robustness of the tree, we carried out a bootstrap analysis with 1000 replicates [45,46].

### 4.3. CRISPR/Cas9-Mediated Mutagenesis

#### 4.3.1. Synthesis of *OfOsp* sgRNA In Vitro

Two target sites were selected on exons 1 and 4 of *OfOsp*. The length of all sgRNA target sequences was 20 base pairs, and their specificity was confirmed through sequencing. sgRNA was subcloned and ligated into a pJET1.2 vector (ThermoFisher Scientific, USA), with the protospacer adjacent motif (PAM) sequence located upstream. sgRNA was synthesized in vitro using a MEGAScript T7 Kit (Ambion, Austin, TX, USA). TrueCut^TM^ Cas9 protein (Invitrogen, Carlsbad, CA, USA) was purchased and stored at −80 °C.

#### 4.3.2. Microinjection of sgRNAs and Detection of OfOsp Mutagenesis

To collect eggs for microinjection, the newly emerged *O. furnacalis* adults were sexed and paired in a plastic bag. Eggs were collected within 1 h of being laid for microinjection under a microscope (Olympus ZSX16, Tokyo, Japan). The microinjection system used in this study was FemtoJet-4i (Eppendorf, Hamburg, Germany) under a microscope (Olympus ZSX16, Tokyo, Japan). Borosilicate Glass Capillaries, with an inner diameter of 0.58 mm, an outer diameter of 1.0 mm, and a length of 10 cm (World Precision Instruments, Sarasota, FL, USA), were used as the needle to deliver a mixture of 300 ng/μL Cas9 protein and 300 ng/μL sgRNA. After injection, eggs were incubated at 28 °C for about 2 days until hatching and later transferred to sterilized containers supplemented with an artificial diet containing yeast extract, wheat bran, vitamin C, sucrose, and agar [38]. All insects were kept under the same rearing condition as described above.

To verify *Osp* knockout in *O. furnacalis*, pupal shells were collected for sequence analysis. Specifically, genomic DNA was extracted using the phenol –chloroform method and precipitated with sodium acetate isopropanol in conjunction with proteinase K (ThermoFisher Scientific, Waltham, MA, USA). Primers targeting the flanking regions of the two target sites were designed to detect gene knockout, and amplification was performed using Hieff Canace Gold High-Fidelity DNA Polymerase (Yeasen, China). Reaction conditions were as follows: pre-denaturation at 98 °C for 3 min; 35 cycles of denaturation at 98 °C for 10 s each, annealing at 55 °C for 20 s, and extension at 72 °C for 30 s; and final extension at 72 °C for 30 s. PCR products were then ligated into a pJET1.2 vector (ThermoFisher Scientific, Waltham, MA, USA) and sent for sequencing by the Genewiz Biotech Company (Suzhou, China).

### 4.4. Phenotypic Observation after Mutagenesis

The phenotypic impact of *OfOsp* knockout on the morphology of virgin adults was observed through dissection using a micro-imaging system (KEYENCE VHX7000, Osaka, Japan). The number of injected eggs, hatching rate of eggs, pupation rate, mutation rate, and sex ratio of eclosed adults were also recorded and are summarized in Table 2.

To investigate whether mutagenesis of *Osp* interferes with the fecundity and fertility of *O. furnacalis*, two-day old virgin *OfOsp* mutant adults were collected and paired with the opposite-sex wildtype adults. Specifically, wildtype females were paired with wildtype males, female *OfOsp* mutants were paired with wildtype males, and male *OfOsp* mutants were paired with wildtype females. Each *O. furnacalis* pair was placed in a transparent PVC bag to allow for mating and oviposition. During this period, eggs laid by each pair were observed and recorded on a daily basis for a 10-day assay period. The hatching rate was recorded for each pair as well. Experiments were performed in three replicates with 5–20 pairs per treatment.

### 4.5. Real-Time Quantitative PCR (RT-qPCR)

To study the expression levels of *OfOsp* across different developmental stages and body parts of *O. furnacalis*, total RNA was extracted from eggs, larvae at instars 1 to 5, female and male pupae, and female and male adults using Trizol reagent (Invitrogen, USA). For spatial expression analysis, tissue was collected from heads, thoraxes (with no legs and wings), legs, forewings, hindwings, abdominal epidermis, fat bodies, ovaries, and external genitalia, and total RNA was extracted. A PrimeScript^TM^ Reverse Transcription Kit (Takara, Japan) was used to synthesize cDNA from 2 μg of total RNA as the template. Quantitative real-time PCR using *OfOsp* gene-specific primers (Table 1) was performed to examine the spatiotemporal expression patterns of *OfOsp*, and *OfRPS3* (GenBank: EU275206.2), a suitable reference gene for *O. furnacalis,* was used as the internal control [47]. The reaction conditions included pre-denaturation at 95 °C for 30 s, followed by 40 cycles of denaturation at 95 °C for 5 s each, annealing at 58 °C for 30 s, and extension at 72 °C for 30 s.

To investigate if *OfOsp* mutagenesis interferes with the transcript levels of other genes, including *Pipe* (GenBank: XM_028307755.1), *Vg* (GenBank: QIH04838.1)*,* and *VgR* (GenBank: QIG55527.1), total RNA was extracted from wildtype *O. furnacalis* and mutant individuals using Trizol reagent (Invitrogen, Carlsbad, CA, USA). According to the manufacturer’s instructions, 2 μg of total RNA was used as the template, and cDNA was synthesized using a PrimeScript^TM^ Reverse Transcription Kit (Takara, Japan). The relative gene expression was calculated following the 2^-ΔΔCq^ method [48].

### 4.6. Statistical Analysis

Statistical analysis of all data was performed using IBM SPSS statistics 22 using a two-tailed *t*-test. Data were represented as the means ± SEM in all cases. *p* < 0.05 was considered a significant difference between treatments and controls.

## 5. Conclusions

In this study, the functionality of *OfOsp* was characterized using CRISPR/Cas9-mediated mutagenesis. Our results support our hypothesis that *OfOsp* is vital for ovarian development and oogenesis in *O. furnacalis*. Given its role in reproduction and the female-specific nature of its mutagenesis, *OfOsp* represents a potential molecular target for future SIT-based control of this global maize pest.

## Figures and Tables

**Table 1:** Primers used in this study.

Primer Name	Primer Sequence (5’-3’)
	Preparation of sgRNA templates
*OSP*_SITE1_F	TAATACGACTCACTATAGGATGCAATTGGATACGGTGGTTTTAGAGCTAGAAATAGCAA
*OSP*_SITE2_F	TAATACGACTCACTATAGGATACTTCGATTGCCCTTTGTTTTAGAGCTAGAAATAGCAA
R80	AAAAGCACCGACTCGGTGCCACTTTTTCAAGTTGATAACGGACTAGCCTTATTTTAACTTGCTATTTCTAGCTCTAAAA
	Identification of mutations
*OFOSP*-SITE1-checkF	TGACACTGGCTTTGTAATGA
*OFOSP*-SITE1-checkR	ATATCCTGTGTTTGGAGCAT
*OFOSP*-SITE2-checkF	TGGAAAGTGATATGAGCAGA
*OFOSP*-SITE2-checkR	TTACGGGATTATTGTGAAGG
*OSP*_FULL_checkF2	AGGACAAGCCAAGCAAAG
*OSP*_FULL_checkR2	TTCCAAGCGATCAAGAGT
	RT-qPCR
*Osp*_Q_F	TGGCTGATCTTCGTGGTCTT
*Osp*_Q_R	CCCGTCATGCTTATTGGCTC
*OFVG1*-F1	CTTCTACCCCACCCACATGT
*OFVG1*-R1	ACCATTTGTCTGCGGAGGTA
*OFVgR*-F1	AGACGACTGTGTAATGGCCA
*OFVgR*-R1	ATCGTCGCAGTCCTCATGAT
*Pipe-F*	AGACGCTGTTCTTCTGTGGA
*Pipe-R*	TGTGTTCAGCTCCTCCCAAT
*Osf-RPS3*-F1	CTGTACGCTGAGAAAGTCGC
*Osf-RPS3*-R1	AACTTCATCGACTTGGCACG
	Cloning of *OfOsp*
*Osp-F1-225*	GCATCCTGTAGTGGTACCTGA
*Osp-R1-1775*	CGGTGATTCTGCTCTTGTGG
*Osp-F2-1413*	CGGAAGAAACAAGCGCTTTC
*Osp-R2-3116*	ACATTGCTCTCACCAGTAATCT
*Osp-F4-3094*	AATGTAGATGACGAGAGTG
*Osp-R4-5402*	AAGCCCAATTCCCTGCAAA
*Osp-F5-4056*	TCTATTGTACAGCCGAGCAGT
*Osp-R5-5535*	CGTGGCTGTTTCTAAGTTTC

**Table 2:** Statistics of *Osp* knockout in *O. furnacalis*.

sgRNA	Injected	Hatched	Pupate (F/M)	Adult (F/M)	Mutant	Mutation Rate
OfOsp	347	32 (61.64%)	134 (59.82%)	95 (38/57)	27	28.42%
GFP	431	384 (89.1%)	147 (76/71)	130 (67/63)	0	0

## Data Availability

All the data and resources generated for this study are included in the article and the Supplementary Materials.

## Supplementary Materials

The following supporting information can be downloaded at https://www.mdpi.com/article/10.3390/ijms242216311/s1: Figure S1: Phylogenetic analysis of Osp amino acid sequences in multiple insect species.
