# Supplementary material for: Knockout of ovary serine protease Leads to Ovary Deformation and Female Sterility in the Asian Corn Borer, Ostrinia furnacalis
Source: Int J Mol Sci. 2023 Nov 14;24(22):16311. doi: 10.3390/ijms242216311 (PMC10671606; doi:10.3390/ijms242216311)
Supplement: Supplementary file 1 [file ijms-24-16311-s001.zip › ijms-2680881-supplementary/ijms-2680881-supplementary.pdf]

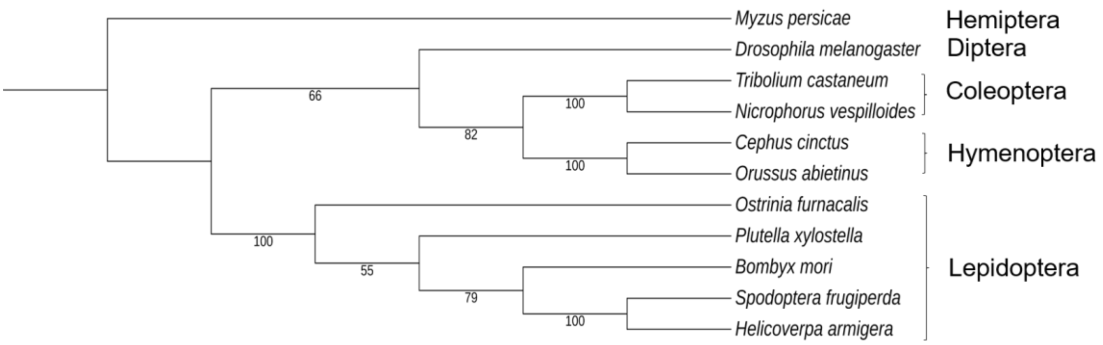

Figure S1. Phylogenetic analysis of Osp amino acid sequence in multi-species. The evolutionary histories of Osp were inferred using the neighbor-joining method. Note: GenBank accession numbers are as follows: *B. mori*（NP_001037168.1）, *H. armigera*（XP_021187246.1）, *P. xylostella*（XP_011552048.1）, *S. frugiperda*（XP_035438721.2）, *D. melanogaster*（NM_079223.2）, *O. abietinus*（XP_012280703.1）, *C. cinctus*（XP_015603565.1）, *N. vespilloides*（XP_017777496.1）, *T. castaneum*（XP_015840900.1）, *M. persicae*（XP_022174293.1）. The OfOsp protein is tightly clustered in the lepidopteran clade (Figure S1), indicating its conserved domain and high sequence identity to OfOsp protein from lepidopteran species.
